# Omega-3 Polyunsaturated Fatty Acids in Cancer Cachexia and Inflammation: Mechanisms and Delivery Innovations

**DOI:** 10.3390/nu18213464

**Published:** 2026-10-22

**Authors:** Marco Cintoni, Elena Leonardi, Elisabetta Meo, Pauline Celine Raoul, Marta Palombaro, Emanuele Rinninella, Lucia Parroni, Esmeralda Capristo, Antonio Gasbarrini, Maria Cristina Mele

**Affiliations:** 1 UOC di Nutrizione Clinica, Dipartimento di Scienze Mediche e Chirurgiche, Fondazione Policlinico Universitario A. Gemelli IRCCS, Largo A. Gemelli 8, 00168 Rome, Italy; marco.cintoni@unicatt.it (M.C.); elena.leonardi2@unicatt.it (E.L.); paulineceline.raoul@policlinicogemelli.it (P.C.R.); marta.palombaro@policlinicogemelli.it (M.P.); emanuele.rinninella@unicatt.it (E.R.); 2 Centro di Ricerca e Formazione in Nutrizione Umana, Università Cattolica del Sacro Cuore, 00168 Rome, Italy; antonio.gasbarrini@unicatt.it; 3 UOC Farmacia, Fondazione Policlinico Universitario A. Gemelli IRCCS, Largo A. Gemelli 8, 00168 Rome, Italy; elisabetta.meo@guest.policlinicogemelli.it (E.M.); lucia.parroni@policlinicogemelli.it (L.P.); 4 Dipartimento di Medicina e Chirurgia Traslazionale, Università Cattolica del Sacro Cuore, 00168 Rome, Italy; esmeralda.capristo@unicatt.it; 5 UOS Medicina della Grande Obesità, Dipartimento di Scienze Mediche e Chirurgiche, Fondazione Policlinico Universitario A. Gemelli IRCCS, Largo A. Gemelli 8, 00168 Rome, Italy; 6 UOC Medicina Interna e Gastroenterologia, Dipartimento di Scienze Mediche e Chirurgiche, Fondazione Policlinico Universitario A. Gemelli IRCCS, Largo A. Gemelli 8, 00168 Rome, Italy

**Keywords:** omega-3 fatty acids, EPA, DHA, cancer cachexia, inflammation, tumor microenvironment, nanoformulations, parenteral nutrition, bioavailability, lipid emulsions

## Abstract

Cancer-associated cachexia and chronic systemic inflammation remain among the most challenging complications in oncology, impairing prognosis, treatment tolerability, and survival. This narrative review examines the mechanistic rationale and clinical evidence for omega-3 polyunsaturated fatty acids (PUFAs), principally eicosapentaenoic acid (EPA) and docosahexaenoic acid (DHA), as adjunctive nutritional and metabolic support agents in this setting, drawing on randomized controlled trials, systematic reviews, meta-analyses, and mechanistic studies, alongside the oral, enteral, parenteral, and nanotechnology-based platforms used to deliver these fatty acids. In experimental models, predominantly cancer cell lines and tumor-bearing rodents, EPA and DHA act on multiple oncological processes, including suppression of pro-inflammatory cytokine synthesis, remodeling of the tumor microenvironment, inhibition of tumor cell proliferation, induction of apoptosis, and attenuation of skeletal muscle catabolism via the ubiquitin-proteasome system and the IGF-1/PI3K/Akt/mTOR axis; in patients, the most consistent mechanistic correlate is a reduction in circulating inflammatory biomarkers. Bioavailability varies substantially by molecular form, with re-esterified triglycerides outperforming ethyl esters and emerging micellar and phospholipid nanoformulations achieving markedly higher absorption in pharmacokinetic and preclinical studies. In advanced non-small cell lung cancer with established cachexia, a meta-analysis of small randomized trials suggests that at least 2 g EPA+DHA per day for six weeks or more may yield modest gains in body weight and quality-of-life scores; this signal, however, derives mainly from pooled analyses and has not been consistently reproduced in individual trials, including large negative trials in mixed cancer populations. Across broader oncological populations, reduction in inflammatory biomarkers is the most consistent finding, effects on chemotherapy tolerability are suggestive but of low certainty, and effects on lean mass and overall survival remain inconclusive. Consistent with ESPEN and ESMO guidance, omega-3 PUFAs should be regarded as metabolic and nutritional support within multimodal cachexia care, not as an anticancer treatment. Omega-3 PUFAs are a biologically grounded and potentially useful, but not yet clinically established, intervention in oncological nutrition, but adequately powered trials with harmonized endpoints, standardized formulations, and stratification by cancer type and cachexia stage are still needed to translate this mechanistic signal into definitive therapeutic protocols.

## 1. Introduction

Cancer remains the second leading cause of mortality worldwide, accounting for nearly 10 million deaths annually [1]. Beyond the direct damage inflicted by malignant transformation, cancer’s systemic consequences, chronic inflammation and cachexia above all, take a heavy and often underestimated toll on survival and treatment outcomes [2].

Cachexia is a multifactorial metabolic syndrome defined by progressive, involuntary loss of skeletal muscle mass, with or without fat depletion, and it cannot be fully reversed by nutritional support alone [3]. Depending on tumor type and stage, it affects 50–80% of cancer patients and accounts directly for up to 22% of cancer-related deaths. The syndrome emerges from a pathological interplay between tumor-derived factors, including proteolysis-inducing factor (PIF) and lipid-mobilizing factor (LMF), and host immune mediators that drive accelerated protein turnover and energy wasting [4,5].

Chronic low-grade inflammation sits at the center of this cascade. Elevated circulating concentrations of TNF-α, IL-6, and IL-1β sustain the catabolic state: they promote tumor cell survival, impair skeletal muscle anabolism, and activate acute-phase hepatic responses that further deplete protein reserves. This inflammatory milieu does not simply follow disease progression, but it helps drive it [6]. The metabolic reprogramming of cachexia also extends well beyond simple undernutrition. Altered insulin signaling, mitochondrial dysfunction, and dysregulated lipid oxidation together create an environment hostile to muscle maintenance [7], and standard caloric supplementation, without addressing the underlying inflammatory and catabolic signals, is rarely enough to reverse or stabilize these losses. Effective intervention requires agents capable of targeting the syndrome’s molecular underpinnings [8].

The relationship between nutritional status and oncological outcomes is well established, yet it still receives too little attention in clinical practice. Malnutrition, present in 30–85% of patients at diagnosis depending on tumor site and stage, is independently associated with increased treatment toxicity, reduced response to chemotherapy and radiotherapy, higher surgical complication rates, and diminished overall survival [9].

Nutritional intervention in oncology has evolved from a reactive, supportive discipline into an active component of integrated cancer care. Current ESPEN guidelines recommend systematic nutritional screening at diagnosis and individualized dietary counseling; where oral intake is insufficient, they recommend stepwise supplementation with oral nutritional supplements (ONS), enteral nutrition (EN), or parenteral nutrition (PN), in that order of preference. Omega-3 PUFAs belong to a broader class of immunonutrients, compounds that modulate immune and inflammatory function beyond simply providing energy or structural substrates, and they have been studied extensively because they act directly on the pathways linking inflammation, immune evasion, and muscle catabolism [10]. This builds on an anti-inflammatory profile first characterized outside oncology, in cardiovascular disease [11].

Omega-3 long-chain PUFAs, principally EPA (C20:5n-3) and DHA (C22:6n-3), with α-linolenic acid (ALA, C18:3n-3) as their plant-derived precursor, are essential fatty acids that the human body cannot synthesize on its own. Marine sources, particularly fatty fish and fish oil concentrates, provide most of the preformed EPA and DHA in the diet; ALA, meanwhile, is found in linseed, chia, and walnuts. The body’s own conversion of ALA to EPA and DHA is quite limited, with efficiencies below 10% for EPA and substantially lower still for DHA [12].

EPA and DHA exert their biological effects through three mechanistically distinct classes of action. As phospholipid membrane constituents, they alter membrane fluidity and lipid raft organization, which in turn modifies transmembrane receptor signaling. They also competitively displace arachidonic acid as a substrate for cyclooxygenase (COX) and lipoxygenase (LOX) enzymes, shifting eicosanoid production away from pro-inflammatory series-2 prostaglandins and series-4 leukotrienes and toward their less inflammatory series-3 and series-5 counterparts, an effect documented for both marine- and plant-derived omega-3 sources [13]. Finally, they serve as precursors of specialized pro-resolving mediators (SPMs), namely resolvins, protectins, and maresins, which help resolve inflammation without causing immunosuppression [14,15].

In oncology, these properties translate into a spectrum of potentially therapeutic actions, demonstrated largely in experimental models: suppressing pro-tumorigenic cytokines, remodeling the TME, inducing cancer cell apoptosis, inhibiting angiogenesis, and slowing the proteolytic pathways that drive cachexia. How much of this translates clinically depends on achieving adequate systemic exposure, which in turn depends on molecular form, route of administration, and formulation technology [16].

This review offers a critically appraised synthesis of current evidence on omega-3 PUFAs in oncology, moving from molecular mechanisms to clinical applications and delivery technologies. Section 2 examines anti-tumor and anti-cachexia mechanisms at the cellular and molecular level (summarized in Table 1 and Figure 1); Section 3 covers routes of administration, bioavailability determinants, and innovative formulation platforms; Section 4 reviews clinical trial data, including RCTs and meta-analyses, distinguishes consistent from inconclusive findings, and stratifies the evidence by fatty acid, dose, formulation, duration, cancer type, and cachexia definition; and Section 5 draws together current knowledge and outlines priorities for future research.

The present work is a narrative review; it does not follow a pre-registered systematic protocol and does not aim to replace the quantitative synthesis provided by the meta-analyses cited throughout. The literature search was conducted in PubMed/MEDLINE and the Cochrane Central Register of Controlled Trials, supplemented by manual screening of the reference lists of key reviews and meta-analyses retrieved. Searches combined MeSH terms and free-text keywords across the domains addressed: (omega-3 OR EPA OR DHA OR polyunsaturated fatty acids) AND (cancer OR neoplasm OR oncology) AND (cachexia OR inflammation OR tumor microenvironment OR clinical trial OR bioavailability OR nanoparticle OR parenteral nutrition). No language restriction was applied; the search covered publications indexed up to May 2025. Priority was given to randomized controlled trials, systematic reviews, and meta-analyses for clinical outcomes; mechanistic evidence was drawn from preclinical studies and pharmacokinetic analyses selected for methodological rigor and relevance to the questions examined. Studies were included on the basis of their direct relevance to one or more sections of the review and the quality of their methodology, as appraised by the authors. Given the narrative design, no formal quality-scoring instrument was applied, and the findings are discussed with explicit reference to the methodological limitations of each body of evidence. To keep mechanistic and clinical evidence clearly separate, each finding is attributed to its source: in vitro studies, animal models, human mechanistic or biomarker data, individual clinical trials, or meta-analyses (Table 1; Section 4.4). Clinical findings were further classified as consistent (concordant direction of effect across trials and pooled analyses), suggestive (favorable pooled estimates not consistently reproduced in individual trials, or data restricted to specific populations), or inconclusive (conflicting, null, or insufficient data).

## 2. Anti-Tumor and Anti-Cachexia Mechanisms of Omega-3 Action

Unless otherwise specified, the mechanisms described in this section derive from in vitro studies in cancer or immune cell lines and from tumor-bearing or cachectic rodent models; the experimental model supporting each mechanism is indicated in the text and in Table 1. Only part of this evidence has been corroborated in patients, chiefly the reduction in circulating inflammatory mediators, and none of these mechanisms has yet been shown to translate by itself into a consistent clinical benefit. The correspondence between mechanistic and clinical evidence is summarized in Section 4.4.

### 2.1. Modulation of Inflammation

The anti-tumor and anti-cachexia properties of omega-3 fatty acids are largely rooted in their ability to suppress pro-inflammatory cytokines, particularly TNF-α and IL-6, whose persistent elevation sustains the catabolic and tumor-permissive milieu described above. EPA and DHA reduce TNF-α and IL-6 synthesis in cultured endothelial and immune cells [13] and, in patients with lung cancer, lower circulating TNF-α, IL-6, and CRP in small RCTs [18,19], dampening both inflammatory signaling and its downstream metabolic consequences [11,13,42,43]. Mechanistically, and on the basis of cellular models, these effects converge on two complementary intracellular targets: omega-3 fatty acids inhibit NF-κB activity while activating PPAR-γ, shifting the cytokine profile away from a pro-inflammatory phenotype [17,18,37]. The omega-6-to-omega-3 ratio matters clinically in its own right, since high dietary omega-6 intake amplifies inflammation and is linked to elevated TNF-α and IL-6; this is part of the rationale for increasing omega-3 intake to rebalance the ratio [12].

The tumor microenvironment (TME) is a complex ecosystem of tumor, stromal, and immune cells, one in which chronic inflammation facilitates immune evasion and promotes angiogenesis [44,45]. Omega-3 fatty acids modify this profile by altering macrophage behavior, reducing IL-6 and TNF-α expression, and shifting the TME toward a less pro-inflammatory state [21,22]; this evidence derives from in vitro and murine studies, and direct evidence in human tumors is lacking. SPMs derived from EPA and DHA, resolvins and protectins among them, help resolve the inflammatory signaling that would otherwise sustain tumor progression [20], although the seminal evidence comes from murine models of acute, non-oncological inflammation. Omega-3 fatty acids also modulate macrophage polarization in experimental models; however, the net effect of this modulation within tumors remains uncertain, because M2-like tumor-associated macrophages can themselves support tumor growth, and their incorporation into cell membrane phospholipids reduces inflammatory mediator production at the cellular level, contributing to a microenvironment less hospitable to tumor growth and metastasis [46,47].

### 2.2. Direct Effects on Tumor Cells

In cancer cell lines and xenograft models, omega-3 fatty acids impede tumor cell proliferation by disrupting key signaling pathways, particularly the PI3K/AKT axis, which is frequently hyperactivated in cancer [26,48]. In human breast cancer cell lines (e.g., MCF-7), EPA and DHA decrease the proliferation index and suppress cyclin D1 expression, producing dose- and time-dependent cell cycle arrest [23,24,25].

Angiogenesis, essential for sustained tumor growth and metastatic spread, is suppressed in endothelial cell cultures and rodent tumor models primarily through inhibition of vascular endothelial growth factor (VEGF). Reduced endothelial cell proliferation and capillary formation limit oxygen and nutrient delivery to tumors, inducing a state of metabolic deprivation that constrains malignant expansion [25,27]. In patients, a reduction in circulating VEGF has been reported in a single small phase II study [28].

One of the most consistently documented direct effects of omega-3 fatty acids on tumor cells in vitro is their ability to induce apoptosis with comparatively lower toxicity towards non-transformed cells in some experimental comparisons, acting through both extrinsic and intrinsic pathways, as shown, for example, in glioblastoma cell models [29]. EPA and DHA increase reactive oxygen species (ROS) generation within tumor cells, leading to oxidative stress and caspase activation [30,49]. They also remodel the Bcl-2 family protein balance, upregulating pro-apoptotic Bax and downregulating anti-apoptotic Bcl-2, which lowers the apoptotic threshold in cancer cells, including prostate cancer cell lines [31,48].

The relationship between omega-3 fatty acids and autophagy is mechanistically complex, given autophagy’s context-dependent role in cancer. EPA and DHA can induce autophagy through modulation of the mTOR pathway [23,24], and DHA has been shown to suppress mTOR expression in MCF-7 breast cancer cells specifically, linking omega-3-mediated autophagic flux to suppression of the PI3K/Akt/mTOR pathway [32]. Apoptosis and autophagy induced together may offer synergistic therapeutic benefit, remodeling the TME while enhancing apoptotic sensitivity at the same time [31,49]. Importantly, the concentrations of free EPA and DHA used in many in vitro studies are not directly comparable to those achieved in human plasma with oral supplementation, and none of these anti-tumor effects has been demonstrated as a clinical endpoint in patients; they should therefore be interpreted as hypothesis-generating.

### 2.3. Sensitization to Conventional Therapies

Experimental studies suggest that omega-3 fatty acids may enhance the efficacy of chemotherapy and radiotherapy while reducing treatment-related toxicity [38]. Lowering levels of prostaglandin E2 (PGE2), a major inflammatory mediator elevated in cancer cachexia, may create a systemic environment more conducive to chemotherapy action [18,35,42,50]. Clinical data are limited: small studies, mostly in lung and breast cancer, have reported improved progression-free survival and fewer side effects, including nausea and fatigue [18,51,52,53], but these findings derive largely from small, often open-label trials and have not been confirmed in adequately powered RCTs (Section 4). For radiotherapy, two independent studies found that topical application of omega-3-rich emulsions reduced the incidence and severity of radiation-induced mucositis, a debilitating complication of head and neck radiotherapy [39,40]. Together these findings suggest that omega-3s may improve radiotherapy’s therapeutic index by protecting normal tissue from radiation injury, which in turn supports patient adherence to treatment [42,50].

A newer area of preclinical interest concerns the interaction between omega-3 fatty acids and immune checkpoint inhibitors (ICIs). In murine cancer models, dietary omega-3 PUFA supplementation enhanced the anti-tumor activity of ICIs, partly by resolving the pro-inflammatory cytokine response that ICIs induce within the tumor microenvironment [54]. This supports omega-3 fatty acids as a biologically plausible adjunct to immunotherapy, though the evidence so far is limited to preclinical models; clinical trials are still needed before this nutrition-immunotherapy interaction can inform actual treatment protocols.

### 2.4. Counteracting Cachexia

In experimental cachexia models, EPA and DHA attenuate cancer cachexia through several converging mechanisms that target metabolism, energy balance, and skeletal muscle protein homeostasis [55]. The anti-inflammatory environment they establish may improve energy balance by boosting mitochondrial efficiency and oxidative capacity in skeletal muscle, favoring substrate utilization patterns that spare lean tissue [35,36,56,57]. At the molecular level, in tumor-bearing mice (MAC16 adenocarcinoma model [33]; orthotopic NSCLC model [34]), omega-3 fatty acids downregulate the ubiquitin-proteasome pathway by reducing MuRF1 and Atrogin-1 expression, suppress NF-κB and myostatin, and boost anabolic signaling through the IGF-1/PI3K/Akt/mTOR axis to promote muscle protein synthesis [35,36,50]; this anabolic effect has also been documented in the exercise-physiology literature [37]. EPA and DHA further promote mitochondrial biogenesis through PGC-1α upregulation in experimental models, helping maintain muscle fiber integrity under catabolic stress [52,53]. Direct confirmation of these molecular effects in skeletal muscle of patients with cancer cachexia is scarce.

It has been proposed that the therapeutic yield depends on cachexia stage, with lean mass preservation expected to be more pronounced in pre-cachectic or early cachexia, while advanced refractory cachexia typically calls for concurrent nutritional and activity-based interventions. This hypothesis, however, has not been tested prospectively in trials stratified by cachexia stage. Clinically, omega-3-enriched regimens have been linked in some studies to stabilization or modest gains in lean mass and body weight, improved body composition, and better tolerance of cancer therapies [36,40,56,57], but these findings are inconsistent across trials and meta-analyses (Section 4.4).

## 3. Routes of Administration and Bioavailability

### 3.1. Route Prioritization: Oral, Enteral, and Parenteral Nutrition

The governing principle in route selection is straightforward: oral feeding should be prioritized whenever the patient retains enough intake capacity. It maintains gastrointestinal motility and mucosal barrier integrity, lowers the risk of enteric infections, and carries substantially fewer complications than parenteral nutrition [58,59,60]. Nutritional counseling is the first-line intervention, with supplementation strategies layered on according to clinical need [61,62].

When oral intake is insufficient, EN becomes the preferred alternative, given its lower complication rates and its capacity to maintain gut mucosal function. It is appropriately reserved for patients who are malnourished or expected to have inadequate oral intake for more than seven consecutive days [62,63]. In one randomized study, a high-protein, *n*-3 PUFA-enriched high-energy ONS produced clinically meaningful improvements in body weight and muscle mass in malnourished cancer patients undergoing chemotherapy, compared with an isocaloric control [60]. Parenteral nutrition (PN), in turn, is recommended by both ESPEN and ASPEN for cancer patients with malnutrition expected to have inadequate energy intake for more than seven days, once oral supplementation and EN prove infeasible or ineffective [58,61,64,65,66].

### 3.2. Bioavailability of Oral Omega-3 Formulations

Oral omega-3 preparations differ markedly in bioavailability depending on their chemical form (Table 2). Oral omega-3 preparations differ markedly in bioavailability depending on their chemical form. Natural triglycerides (TG), the form found in dietary fish, show good intestinal absorption. Pharmacological evaluation of lipid absorption, however, has moved well past these traditional standards. Advanced micellar and phospholipid delivery systems have been reported, in small pharmacokinetic studies, to achieve up to 10 to 11 times the bioavailability of standard ethyl ester or natural triglyceride oils. These platforms reduce the need for high-fat co-ingestion and are less dependent on standard lipase-dependent pathways; whether this advantage is preserved in patients with cancer or with compromised gastrointestinal function has not yet been established.

Phospholipid-bound omega-3s, as found in krill oil, show bioavailability equal to or exceeding that of TG. Ethyl esters (EE), produced through industrial concentration and transesterification, show comparatively poorer absorption, particularly under fasted conditions [16,67].

Re-esterified triglycerides (rTG) achieve the highest intestinal absorption of any oral form, since they serve as optimal substrates for pancreatic lipase hydrolysis and micellar incorporation. Dietary fat intake alongside them is important to activate bile and lipase secretion; proton pump inhibitors, H2-receptor antagonists, and excessive dietary fiber can all impair absorption [16]. This interaction has a practical corollary. When absorption is limited, for example, by a high dietary fiber intake, a fat-restricted diet, or malabsorption, clinicians may be tempted to compensate by escalating the dose of EPA and DHA. High-dose omega-3 PUFAs exert mild antiplatelet effects and may potentiate the action of the anticoagulant and antiplatelet drugs widely used in oncology, such as low-molecular-weight heparins, direct oral anticoagulants, and vitamin K antagonists prescribed for the prevention or treatment of cancer-associated thrombosis. A meta-analysis of large cardiovascular RCTs did not show a significant overall increase in bleeding events with omega-3 supplementation, but a higher bleeding risk was observed in trials of high-dose purified EPA [68]; moreover, patients with cancer often carry additional bleeding risk factors, such as chemotherapy-induced thrombocytopenia, hepatic dysfunction, or mucosal lesions. Rather than empirically increasing the dose, it is therefore preferable to optimize formulation and timing (e.g., rTG or phospholipid forms, intake with a fat-containing meal, separation from high-fiber meals) and when high doses (e.g., >3 g/day) are combined with anticoagulant or antiplatelet therapy or used in patients with thrombocytopenia to monitor platelet count, coagulation parameters, and clinical signs of bleeding. Some formulation technologies improve bioavailability without needing high-fat co-ingestion at all, which carries real clinical value: a self-microemulsifying drug delivery system (SMEDDS) built around high-concentration omega-3 EE achieves rapid spontaneous emulsification on contact with gastrointestinal fluids, substantially improving absorption under fasted conditions [67,69]. An L-lysine salt formulation of EPA and DHA takes a different route, exploiting acid dissociation in the stomach to release free fatty acids for direct small intestinal absorption, without relying on lipase-mediated hydrolysis at all [70]. The main oral formulations are compared in Table 2, together with their advantages and limitations

**Table 2:** Oral omega-3 formulations: molecular forms, bioavailability, and clinical characteristics.

Molecular Form	Main Source	Bioavailability	Key Advantage	Limitation
Natural TG	Fatty fish, fish oil	Good (reference)	Physiological absorption via pancreatic lipase	Requires fat co-ingestion for optimal absorption
Phospholipid (PL)	Krill oil	Equal to or >TG	High water dispersibility; no fat co-ingestion needed	Higher cost; lower EPA/DHA concentration per gram
Ethyl Esters (EE)	Industrial concentrate	Lower, esp. fasted	High EPA+DHA concentration per capsule; widely available	Lipase-dependent; markedly reduced absorption without food; absorption further reduced by high-fiber meals—avoid compensatory dose escalation in patients on anticoagulants
Re-esterified TG (rTG)	Industrial re-esterification	Highest of all forms	Optimal lipase substrate; superior absorption fasted and fed	Higher production cost
L-lysine salt (EPA/DHA)	Synthetic salt	Higher than EE and TG	Acid dissociation in stomach; lipase-independent; no fat co-ingestion needed	Limited clinical dataset; not widely available
SMEDDS formulation	EE in self-emulsifying matrix	Substantially > EE	Spontaneous emulsification on GI contact; suitable for low-fat diets	Formulation complexity; limited clinical data

TG = triglyceride; PL = phospholipid; EE = ethyl ester; rTG = re-esterified triglyceride; SMEDDS = self-microemulsifying drug delivery system; EPA = eicosapentaenoic acid; DHA = docosahexaenoic acid; GI = gastrointestinal.

### 3.3. Bioavailability in Cancer Patients and Nanotechnology-Based Delivery Systems

In cancer patients, EPA and DHA bioavailability can vary considerably depending on the chronic inflammatory state and any associated malnutrition or cachexia. Decreases in fat and muscle mass alter lipid metabolism and intestinal fatty acid absorption, a pharmacokinetic dimension that is frequently overlooked in clinical dosing strategies [21,71,72].

To get around the limits of conventional formulations, researchers have developed nanotechnology-based delivery platforms: nanoemulsions, liposomes, solid lipid nanoparticles (SLNs), and polymeric nanocapsules. All of the evidence reviewed in this section comes from preclinical models, in vitro cell lines and animal studies, and no clinical trials have yet evaluated these platforms; whether they will translate to human oncology remains an open question. Within these preclinical models, nanotechnology-based systems have shown enhanced absorption, tumor-selective targeting, and reduced systemic toxicity; the platforms, omega-3 cargoes, targeting mechanisms, tumor models and key outcomes are summarized in Table 3. 

**Table 3:** Nanotechnology-based omega-3 delivery systems: preclinical evidence.

Platform	Omega-3 Cargo	Targeting Mechanism	Tumor Type (Model)	Key Outcome
LDL nanoparticles	DHA	LDL receptor-mediated endocytosis (exploits upregulated receptor in cancer cells)	Hepatocellular carcinoma (in vitro/in vivo)	Selective tumor cell uptake; pro-oxidative cell death induction
Tuftsin-functionalized nanoliposomes	ALA	Tuftsin peptide targets macrophage Fc receptors in tumor microenvironment	Hepatocellular carcinoma (murine model)	Improved survival; dual antitumor and immunomodulatory activity
pH/temperature-responsive liposomes	DHA	Phytanic lipid shell responds to acidic tumor microenvironment	Breast cancer (MCF-7, MDA-MB-231)	Greater apoptosis vs. free DHA; reduced *p*-Akt expression
Solid lipid nanoparticles (SLN)	DHA + doxorubicin	Passive EPR effect; lipid matrix controls co-release	Lung cancer (in vitro)	Encapsulation ~100%; improved cellular uptake; enhanced cytotoxicity vs. monotherapy
DHA-functionalized BSA nanoparticles	Docetaxel (DHA surface)	Phosphatidylethanolamine overexpression on lung tumor cells used as docking site	Lung cancer (in vivo)	Significantly improved anti-tumor efficacy; prolonged survival

DHA = docosahexaenoic acid; ALA = alpha-linolenic acid; LDL = low-density lipoprotein; SLN = solid lipid nanoparticle; BSA = bovine serum albumin; EPR = enhanced permeability and retention. All data are from preclinical models (in vitro or animal studies); no clinical trial evidence is available for these platforms.

DHA-loaded LDL nanoparticles, for instance, can selectively target liver cancer cells by exploiting natural endocytosis mechanisms to induce cell death through pro-oxidative processes [73], while tuftsin-functionalized nanoliposomes encapsulating ALA improved survival in murine hepatocellular carcinoma models, showing dual antitumor and immunomodulatory activity [74].

In breast cancer, DHA encapsulated in pH- and temperature-responsive phytanic lipid liposomes reduced cell viability in MCF-7 and MDA-MB-231 cells and induced apoptosis more effectively than free DHA, while also decreasing *p*-Akt expression. In lung cancer, SLNs containing both doxorubicin and DHA achieved drug encapsulation efficiency approaching 100%, along with better cellular uptake and enhanced cytotoxicity compared with either agent alone [75]. Separately, docetaxel-loaded BSA nanoparticles surface-functionalized with DHA exploited elevated phosphatidylethanolamine expression in lung tumor cells as a targeting mechanism, significantly improving anti-tumor efficacy and prolonging survival in vivo [76]. 

### 3.4. Oral Pharmaceutical Formulations and Treatment Adherence

Oral omega-3 preparations come mainly as soft gelatin capsules, tablets, and powders. Soft capsules remain the predominant delivery system: they protect against lipid oxidation, mask taste and smell, allow precise dosing, and offer formulation versatility. Enteric-coated variants delay release until the intestine’s alkaline environment is reached, improving tolerability for gastric-sensitive patients [16]. Liposomes, nanoemulsion droplets, nanostructured lipid carriers, and microgel matrices represent a rapidly evolving frontier in encapsulation technology [77,78].

Treatment adherence is a critical determinant of efficacy, and it is not evenly distributed: younger patients, unemployed individuals, and smokers are disproportionately represented among those with poor adherence [79]. Digital health interventions, including smartphone applications that combine bidirectional patient–clinician communication with automated reminders, have improved adherence by enabling real-time problem resolution [80,81]. Non-adherence to ONS tends to stem from fatigue, inadequate clinical supervision, and limited social support, and it should be monitored as a multidimensional, longitudinal construct rather than a single fixed trait [16,82].

Conditions causing partial or generalized malabsorption, such as exocrine pancreatic insufficiency, coeliac disease, Crohn’s disease, and gastric or intestinal resections, are common in oncology patients and need to be identified early so that nutritional support can be properly targeted [72,83].

### 3.5. Intravenous Lipid Emulsions: Formulations and Clinical Evidence

Intravenous lipid emulsions (ILEs) are an indispensable component of PN, supplying concentrated energy, essential fatty acids, and fat-soluble vitamins while also modulating immunity and inflammation. Contemporary multi-lipid formulations combine medium-chain triglycerides (MCT), olive oil (OO), and fish oil (FO) to achieve better metabolic and immunological profiles than first-generation soybean oil (SO)-only preparations. The hepatotoxic potential of SO-based ILEs, driven by phytosterols and excess omega-6 PUFAs, stands in contrast to the hepatoprotective properties of FO-based preparations, consistent with omega-3’s broader role in hepatic lipid partitioning [17,70,84].

There is substantial evidence supporting the clinical benefits of PN with fish oil-containing ILEs. A systematic review and meta-analysis of 26 RCTs found that FO-containing PN reduces infectious complications (RR 0.65; 95% CI 0.44–0.95) and shortens ICU length of stay in critically ill patients [85]. Subsequent network meta-analyses have consistently confirmed FO-containing ILEs as the most effective intervention for improving clinical outcomes in hospitalized adults, a position ESPEN itself endorses [14,86]. Table 4 summarizes the principal commercial formulations, their compositions, and their clinical applications. Brand names are retained here because these are proprietary multi-lipid mixtures with no INN generic equivalent, and clinicians choose among them by product; still, it is the composition and omega-6:omega-3 ratio columns that carry the pharmacologically relevant information.

For cancer patients with intestinal failure and a life expectancy beyond 1–3 months, home parenteral nutrition (HPN) is the strategy recommended by ESPEN guidelines [87]. In neuroendocrine tumor patients with intestinal failure, available data show 58% one-year survival after HPN initiation, with low infectious complication rates of 0.5–2 episodes per 1000 catheter-days [88,89,90].

### 3.6. Comparative Analysis: Oral Versus Parenteral Route

Among oral LC-PUFA formulations, absorption variability comes down to physiological factors (food intake, gastric pH, drug interactions) as well as the formulation’s own physicochemical characteristics. Parenteral routes play an indispensable role when oral or enteral intake is impossible or ineffective, since parenteral administration bypasses gastrointestinal transit and first-pass hepatic metabolism entirely, ensuring complete bioavailability and predictable plasma kinetics [70,86].

Fish oil-enriched ILEs, dosed at 0.1–0.2 g/kg/day per ESPEN recommendations, consistently show a more favorable inflammatory and metabolic profile than MCT/SO or OO comparators [14,70]. Most of this evidence, however, comes from critically ill non-oncological patients on total parenteral nutrition; direct evidence in oncological patients is more limited, so any claim of FO-ILE superiority in this specific population should be treated with some caution. Within oncology, the parenteral route is most clearly indicated for patients with severe gastrointestinal dysfunction that precludes enteral absorption, such as after gastrointestinal resection, radiation enteritis, or bowel obstruction, where ILE-based omega-3 delivery ensures complete bioavailability regardless of mucosal integrity [14,86]. Maintaining safety in PN requires interdisciplinary management, automated control procedures, and properly trained healthcare personnel [91]. Figure 2 summarizes the hierarchical route selection framework and the available delivery options.

## 4. Clinical Evidence

Before the clinical data are examined, the therapeutic role of omega-3 PUFAs must be clearly delimited. In accordance with ESPEN guidance, long-chain *n*-3 fatty acids are considered exclusively as a component of nutritional and metabolic care: in patients with advanced cancer undergoing chemotherapy who are at risk of weight loss or malnourished, ESPEN suggests supplementation with long-chain *n*-3 fatty acids or fish oil to stabilize or improve appetite, food intake, lean body mass, and body weight (weak recommendation, low level of evidence), while stating that no diet is known to reproducibly cure cancer or prevent its recurrence [92]. The ESMO clinical practice guideline similarly addresses *n*-3 fatty acids only within the multimodal supportive management of cancer cachexia [39], and the ASCO guideline considered the evidence insufficient to recommend omega-3 fatty acid supplementation for the treatment of cancer cachexia [93]. None of these guidelines recommends EPA or DHA for the treatment of specific cancer types or as agents directed against cancer cells. The anti-tumor effects discussed in Section 2 derive from experimental models and should not be taken to imply that omega-3 supplementation is a form of anticancer therapy; throughout this section, omega-3 PUFAs are considered as metabolic and nutritional support for patients with cancer, particularly those with advanced disease. The clinical evidence is presented by tumor type (Section 4.1, Section 4.2 and Section 4.3), then synthesized by outcome, distinguishing consistent from inconclusive findings (Section 4.4), and finally stratified by the main sources of heterogeneity (Section 4.5).

### 4.1. Evidence in NSCLC-Associated Cachexia

Taken as a whole, the randomized controlled trial (RCT) evidence is heterogeneous (Table 5). 

When the trials are stratified by tumor type, cachexia stage, and primary endpoint, the most favorable, although still limited, signal comes from advanced non-small cell lung cancer (NSCLC) cohorts with established cachexia, a group marked by heavy systemic inflammatory burden, rapid lean mass depletion, and a markedly limited response to conventional nutritional interventions [40]. Weight stabilization and improvements in quality of life have been reported in these NSCLC populations, mainly in small trials [40,41]. Breast cancer cohorts undergoing neoadjuvant chemotherapy tell a different story: the DHA WIN trial found no significant benefit for functional outcomes or quality of life in this group [98].

Within the NSCLC subpopulation, the meta-analysis by Chua et al. is the main pooled analysis available. Pooling a limited number of small trials of patients with advanced NSCLC and cachexia, it found statistically significant increases in body weight along with improvements in health-related quality of life, particularly on the Physical Functioning and Global Health subscales of the EORTC QLQ-C30, whereas lean body mass did not change significantly [40]. Because this estimate rests on few, small, and heterogeneous trials, it should be regarded as hypothesis-supporting rather than confirmatory. These results need to be weighed, though, against the 2024 findings from the Phase III MENAC (Multimodal-Exercise, Nutrition and Anti-inflammatory medication for Cachexia) trial, which showed that multimodal interventions incorporating omega-3 PUFAs stabilize body weight without reversing skeletal muscle wasting [102]. Put together, these findings suggest a possible, but still unconfirmed, role for omega-3 supplementation: useful for arresting further catabolic deterioration, but not, on its own, capable of reversing muscle loss that has already set in [102].

Individual RCTs add further texture to this picture, although they are small and heterogeneous in design. Finocchiaro et al., using a placebo-controlled design, documented a significant reduction in circulating inflammatory and oxidative stress biomarkers during chemotherapy [19]. A double-blind RCT by Cheng et al. (2021), involving 60 NSCLC patients at nutritional risk, found significant improvements in body weight, serum albumin, CRP, and TNF-α relative to placebo after 12 weeks [18]. In a double-blind RCT in 40 patients with stage III NSCLC undergoing chemoradiotherapy, an ONS providing 2.0 g EPA and 0.9 g DHA per day for five weeks better preserved body weight and fat-free mass than an isocaloric control [94]. In the preclinical space, van de Worp et al. (2024) used an orthotopic NSCLC murine model to show that a multi-nutrient intervention including fish oil significantly delayed cachexia onset, reduced loss of both skeletal muscle and adipose tissue, and suppressed molecular markers of ubiquitin-proteasome signaling in skeletal muscle [34].

On posology, a threshold of approximately 2 g of EPA+DHA per day for at least six weeks has been proposed to produce improvements in body weight and quality of life, using conventional lipid formulations [40]. This threshold derives primarily from the subgroup analyses of the Chua et al. meta-analysis, which pooled a small number of trials in advanced NSCLC [40]; it has not been validated prospectively, and comparable or higher doses and durations failed to improve body weight or lean mass in several individual RCTs, including large double-blind trials in mixed or pancreatic cancer populations [95,99,100] and a phase III trial after total gastrectomy [97] (Table 5). The 2 g/day, six-week figure should therefore be regarded as a pragmatic, hypothesis-generating reference derived mainly from meta-analytic evidence, rather than as an established minimum effective dose. The arrival of micellar and phospholipid delivery systems adds a further element of uncertainty. Because these advanced vehicles have been reported to achieve up to 10 to 11 times the intestinal absorption of standard esterified or triglyceride oils, a dose threshold defined for conventional formulations may not be directly transferable to them; whether equivalent systemic exposure, and clinical effect, can be achieved at lower oral doses remains to be tested. Where systemic exposure falls short of that threshold, or where a study runs for too short a time, null results may partly reflect insufficient exposure rather than a true lack of efficacy; however, some negative trials documented increased plasma EPA levels, so this explanation cannot account for all null findings [95,100].

### 4.2. Evidence in Other Tumor Types

Patients with gastrointestinal cancers deserve particular attention. Tumors of the upper gastrointestinal tract and pancreas are associated with the highest prevalence of malnutrition and the most severe weight loss among solid tumors, owing to the combined effects of anorexia, mechanical obstruction, maldigestion and malabsorption, and major surgery [9,103]; in a large French multicenter survey, malnutrition affected about two-thirds of patients with pancreatic cancer and approximately 60% of those with esophago-gastric cancer [103]. Gastrointestinal oncology is therefore the setting in which nutritional support, including EPA/DHA-enriched formulations, has the strongest clinical rationale and the greatest potential absolute benefit. Several trials of EPA-enriched supplements have indeed been conducted in these patients, with some encouraging results, such as preservation of lean body mass with EPA-enriched enteral nutrition after esophagectomy [96] and improved tumor response and inflammatory profile with intravenous fish oil in advanced esophago-gastric cancer [28]. The evidence, however, is not uniformly positive: the largest RCT in pancreatic cancer was negative in the intention-to-treat analysis [95], and a phase III RCT after total gastrectomy found no effect on lean body mass [97]. Thus, patients with gastrointestinal cancers are the group with the greatest nutritional need and potential benefit, but a greater efficacy of EPA/DHA in this population than in other tumor types has not yet been demonstrated in comparative analyses.

The evidence base in gastrointestinal, breast, and head and neck cancers is more scattered, partly because these tumor types cover a wide range of cachexia prevalence and inflammatory profiles, and partly because trials in these populations have rarely used cachexia stage as a stratification variable [104]. Two meta-analyses by Delpino and Figueiredo, pooling 14 trials across pancreatic, gastrointestinal, lung, head and neck, and breast cancer, found that *n*-3 PUFA interventions could improve lean body mass under certain conditions [105,106]. A related paper by the same authors discusses omega-3 supplementation and body composition in cancer more broadly, without a formal pooled analysis [21]. The *I*^2^ values in these meta-analyses were high, and the reasons are not hard to identify: doses ranged from under 1 g to over 4 g EPA+DHA per day, supplementation periods ran from two weeks to six months, baseline nutritional status varied widely, and concurrent treatments were far from uniform. It should also be noted that the largest double-blind RCTs conducted in mixed populations of patients with cancer cachexia, predominantly gastrointestinal and lung cancer, were negative: EPA diester at 2 or 4 g/day for eight weeks did not improve body weight or lean body mass versus placebo in 518 patients [100]; an EPA-enriched supplement was not superior to megestrol acetate, and did not add to it, in 421 patients [99]; and in 200 patients with pancreatic cancer an EPA-enriched ONS did not improve weight or lean mass in the intention-to-treat analysis, although a dose–response relationship with EPA incorporation was observed in compliant patients [95]. Accordingly, a Cochrane review concluded that the data were insufficient to establish that oral EPA is superior to placebo for cancer cachexia [107].

DHA’s evidence base in breast cancer looks somewhat different. Both overall survival and progression-free survival have been reported as improved in DHA-supplemented cohorts in small, mostly non-randomized studies [41,108,109], yet the DHA WIN trial found that DHA supplementation during neoadjuvant chemotherapy made no significant difference to quality of life or exercise behavior relative to placebo [98]. Even a null result like this one is informative here: it suggests that DHA’s previously reported survival and chemosensitization benefits in breast cancer may not extend to patient-reported quality of life or functional outcomes during active treatment. The EORTC QLQ-C30 global scale, after all, reflects a composite of disease-related symptom burden and psychological wellbeing that may be relatively insensitive to nutritional intervention.

Some of the most interesting gastrointestinal evidence comes from trials with explicitly negative results. Aoyama et al. (2019) ran a multicenter phase III RCT in 123 patients undergoing total gastrectomy for gastric cancer and found no significant difference in lean body mass loss between EPA-enriched ONS and a standard diet at one or three months after surgery [97]. One possible explanation for this null finding is that total gastrectomy causes an acute catabolic insult that a short perioperative course of anti-proteolytic EPA signaling is unlikely to offset. Eltweri et al. (2019) found something quite different in a single-arm phase II study (*n* = 20) of intravenous Omegaven^®^ plus EOX chemotherapy in advanced esophago-gastric adenocarcinoma: a partial radiological response rate of 73% versus 39% in historical controls (*p* = 0.03), alongside significant suppression of IL-2, TNF-α, and VEGF [28]; the comparison with historical controls, however, precludes causal inference.

### 4.3. Survival and Quality of Life

A survival benefit from omega-3 supplementation is biologically plausible on several counts: attenuating systemic inflammation reduces tumor-promoting cytokine signaling, and preserving lean mass is independently linked to better chemotherapy tolerability and lower treatment-related mortality. Some reviews have reported an association between omega-3 supplementation and improved overall survival in selected oncological populations [43]. That said, this should be read against the methodological limits of the underlying studies: most were not powered for survival as a primary endpoint, follow-up ranged from weeks to months, and survival was often a secondary or exploratory outcome rather than the main focus. Overall, a survival benefit has not been demonstrated and should be considered inconclusive.

Quality of life data point in a somewhat more favorable, but still heterogeneous, direction. Systematic reviews and meta-analyses report significant associations between *n*-3 PUFA supplementation and improved functional domain scores on the EORTC QLQ-C30, with the Physical Functioning and Global Health subscales affected most often [109,110]; these improvements, however, derive mainly from NSCLC populations [40] and were not observed in the DHA WIN trial in breast cancer [98]. The Fatigue and Appetite Loss subscales, the ones most directly tied to the inflammatory and catabolic mechanisms omega-3s are thought to modulate, come up less consistently across primary trials; this may partly reflect selective outcome reporting, although a true absence of effect cannot be excluded.

### 4.4. Consistent Versus Inconclusive Findings: An Outcome-Based Synthesis

Table 6 summarizes the clinical evidence outcome by outcome, mapping each mechanistic domain described in Section 2 against the corresponding clinical evidence, making explicit where the evidence is still confined to experimental models. Three levels can be distinguished. First, one finding is consistent: reductions in circulating inflammatory biomarkers (CRP, IL-6, TNF-α) have been reported in most trials that measured them and are supported by pooled analyses [18,19,28,42]; this, however, is a surrogate endpoint, and its clinical significance depends on translation into patient-relevant outcomes. Second, several findings are suggestive: modest gains or stabilization of body weight, improvements in selected quality-of-life domains in advanced NSCLC, and fewer chemotherapy-related toxicities are supported by meta-analyses or small trials [28,40,41,101], but were not reproduced consistently in individual RCTs, including the largest double-blind trials [95,99,100]. Third, effects on lean body mass, tumor response, and overall survival remain inconclusive, being conflicting or based on underpowered, non-randomized, or secondary analyses [43,95,97,100,101]. By contrast, the direct anti-tumor mechanisms (inhibition of proliferation, induction of apoptosis and autophagy, anti-angiogenic activity, TME remodeling, and interaction with immune checkpoint inhibitors) have been demonstrated only in cell lines and animal models and have not been assessed as clinical endpoints in patients with cancer cachexia.

### 4.5. Heterogeneity of the Clinical Evidence and Stratified Interpretation

The clinical studies discussed above differ in at least six respects that limit the pooling and generalization of their results (Table 7). First, fatty acid composition: trials tested EPA alone or EPA-predominant supplements [95,96,99,100], DHA alone [98], or combined EPA+DHA preparations [18,19,41,94]; since EPA and DHA differ in incorporation kinetics and in the lipid mediators they generate, and the EPA:DHA ratio may itself influence anti-inflammatory potency [112], results obtained with one preparation cannot be extrapolated to the others. Notably, the largest negative trials used EPA-predominant preparations, whereas the most favorable NSCLC data come mainly from combined EPA+DHA formulations. Second, dose: daily doses ranged from less than 1 g to more than 4 g; a dose–response relationship has been described for body weight only [101], and the only trial that compared two doses head-to-head found no benefit from either [100]. Third, formulation and route: fish oil capsules (ethyl esters or triglycerides), protein- and energy-dense ONS enriched with EPA, EPA diester, enteral formulas, and intravenous fish oil emulsions have all been used; in ONS-based trials the effect of omega-3 fatty acids cannot be fully separated from that of the accompanying energy and protein, and intravenous data derive from small, uncontrolled studies. Fourth, treatment duration: interventions ranged from short perioperative courses to several months, and few trials exceeded 12 weeks. Fifth, cancer type and treatment setting: NSCLC during chemotherapy, gastrointestinal cancers in surgical or palliative settings, breast cancer during neoadjuvant chemotherapy, and mixed populations have been studied, each with a different inflammatory burden and nutritional risk. Sixth, cachexia definition: many trials predate the 2011 international consensus [3] and used heterogeneous weight-loss thresholds, nutritional risk screening, or no nutritional criterion at all, while cachexia stage (pre-cachexia, cachexia, refractory cachexia) was rarely pre-specified.

Reading the evidence along these strata supports a more cautious and more precise clinical interpretation (Table 7). The most favorable signal concerns combined EPA+DHA preparations at ≥2 g/day, given for at least six weeks to patients with advanced NSCLC receiving chemotherapy. EPA-predominant supplements given to mixed or pancreatic cancer populations with established weight loss, and a short perioperative course after total gastrectomy, were largely ineffective on weight and lean mass, with the notable exception of one perioperative trial after esophagectomy [96]. Evidence for DHA alone is limited to breast cancer and does not support a benefit on quality of life. Future meta-analyses should therefore stratify by these factors rather than pool heterogeneous interventions, and future trials should report them explicitly. Consistent with the narrative design of this review, and given the extent of this clinical heterogeneity, we did not attempt a new quantitative pooling, which would yield an average estimate corresponding to no specific preparation or population; the evidence is instead synthesized qualitatively according to these strata.

### 4.6. Methodological Constraints and Research Priorities

Klassen et al. found that while individual trials suggest benefit, the overall body of evidence remains inconclusive, hampered by small samples, heterogeneous regimens, and variable formulations [71]. That is not a reason to give up clinical interest in omega-3 supplementation, though; if anything, it points squarely to what still needs doing. Three structural problems keep recurring in the literature. First, formulation heterogeneity: trials have used EE, rTG, phospholipid-bound preparations, and fish oil concentrates more or less interchangeably, despite bioavailability differences that clearly matter, and pooling them produces an average effect estimate that corresponds to no actual clinical preparation [113]. Second, the EPA:DHA ratio has largely been treated as irrelevant in trial protocols, even though emerging evidence suggests it may affect anti-inflammatory potency independently of total dose [112]. Third, interactions with concurrent chemotherapy have never been systematically characterized, and some cytotoxic regimens may well potentiate the pro-oxidant activity of omega-3s in tumor cells while others remain pharmacokinetically unaffected [38].

Ghoreishy et al. (2024) put a number on this variance in a dose–response meta-analysis of 33 RCTs (*n* = 2047): body weight rose in a dose-dependent way with each 1 g/day increment up to 4 g/day (standardized mean difference [SMD] 0.52 kg; 95% CI 0.31–0.73), while lean body mass, fat mass, BMI, and body fat percentage all failed to reach statistical significance, with *I*^2^ values of 79–97% across every body composition endpoint [101]. This is compatible with the tentative dose threshold discussed in Section 4.1 and suggests that doses approaching 4 g/day may bring additional body-weight benefit, although in the only large RCT that directly compared 2 and 4 g/day of EPA neither dose was superior to placebo [100]. A second dose–response meta-analysis restricted to patients with cancer cachexia also focused on body weight [111]. No study so far has been adequately powered to tell whether this relationship extends to lean body mass or survival, and the optimal EPA:DHA ratio within this dose range remains uncharacterized [112]. What the field really needs are adequately powered, phase III-style trials using CT-based lean body mass as a co-primary endpoint alongside a validated patient-reported outcome instrument, with cachexia stage (defined according to the international consensus [3]) pre-specified as a stratification variable, stratification or restriction by tumor type, a standardized and fully reported formulation (e.g., ≥2 g EPA+DHA/day in a well-characterized molecular form, with the EPA:DHA ratio, vehicle, and route specified), objective verification of exposure and adherence (e.g., plasma or erythrocyte EPA/DHA incorporation), and a minimum follow-up of twelve weeks [12,69].

## 5. Conclusions and Future Perspectives

The evidence reviewed here supports the biological plausibility and potential clinical relevance of EPA and DHA as adjunctive nutritional and metabolic support agents in oncological nutrition. Their mechanisms act on several fronts at once, touching the tumor microenvironment, systemic inflammatory cascades, skeletal muscle proteolytic pathways, and the cancer cell’s own apoptotic machinery, reaching more points in cancer biology than most nutritional interventions manage to. Suppression of TNF-α, IL-6, and IL-1β, the dual modulation of NF-κB and PPAR-γ, the slowing of the ubiquitin-proteasome axis via MuRF1 and Atrogin-1, and the upregulation of the IGF-1/PI3K/Akt/mTOR pathway together describe a coherent anti-cachectic mechanistic framework that derives largely from preclinical models; in patients, only the anti-inflammatory component has been documented consistently.

How well this works clinically depends on dose, but just as much on molecular form, delivery platform, and route of administration. Re-esterified triglycerides beat ethyl esters on oral bioavailability; intravenous fish oil-containing lipid emulsions guarantee complete systemic exposure for patients who cannot absorb lipids enterally; and newer nanotechnology-based platforms, including LDL-based nanoparticles, pH-responsive liposomes, and DHA-functionalized polymeric nanocarriers, point toward tumor-selective delivery that has not yet reached clinical testing.

Clinical findings must be weighed according to their consistency (Table 6). The most consistent finding is a reduction in circulating inflammatory biomarkers. Modest gains in body weight and improvements in selected quality-of-life domains are suggested by meta-analytic evidence, primarily in advanced NSCLC with cachexia at doses of at least 2 g/day for a minimum of six weeks [40], but have not been reproduced consistently across individual RCTs, including large negative trials [95,99,100]. Effects on lean body mass, chemotherapy tolerability, tumor response, and survival remain inconclusive. It is plausible, but not yet demonstrated, that the greatest therapeutic yield is achievable in pre-cachectic or early-stage cachexia, before muscle loss becomes irreversible, and in patients at the highest nutritional risk, such as those with gastrointestinal cancers.

The main limitations of the current evidence base are methodological and translational. Heterogeneity in fatty acid composition (EPA, DHA, or both), patient selection, tumor type, cachexia definition, formulation, dosing, duration, and endpoint reporting has kept a plausible biological signal from translating into definitive clinical guidance. What is needed next are adequately powered RCTs with harmonized primary endpoints, CT-based lean body mass, validated inflammatory biomarker panels, and patient-reported instruments such as the EORTC QLQ-C30, with cachexia stage pre-specified as a stratification variable and supplementation periods of at least twelve weeks. The optimal EPA:DHA ratio still needs dedicated study, since emerging evidence suggests lower ratios may bring greater anti-inflammatory benefit [112]. Pharmacogenomic profiling of FADS1/FADS2 polymorphisms may eventually make individualized dosing strategies possible.

In line with ESPEN and ESMO guidance, omega-3 PUFAs should be considered metabolic and nutritional support for patients with advanced cancer, not an anticancer treatment [39,92]. Omega-3 PUFAs seem to work best not as standalone supplements but as one active component within multimodal anti-cachectic protocols, combined with resistance exercise and adequate protein intake [114]; when high doses are used, potential interactions with anticoagulant and antiplatelet therapy should be considered [68]. Omega-3-based nanoformulations, from DHA-loaded liposomes to surface-functionalized polymeric nanocarriers, are still at the preclinical stage and will need first-in-human phase I trials before their real value can be judged. The underlying biology appears plausible; what happens from here depends on methodological rigor, sound patient stratification, and a willingness to carry the most promising delivery platforms into the clinic.

## Figures and Tables

**Figure 1:**
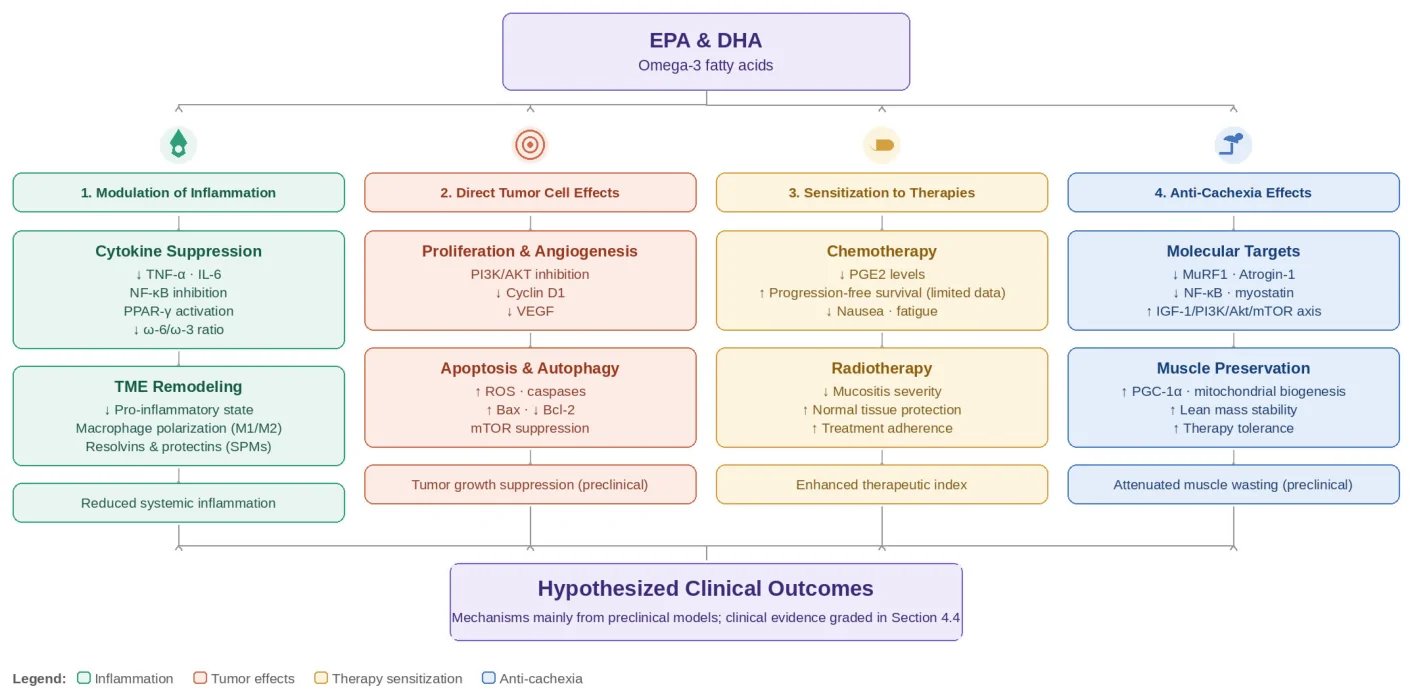
Overview of the anti-tumor and anti-cachexia mechanisms of EPA and DHA in cancer. ↑ upregulation; ↓ downregulation. The mechanisms shown derive predominantly from in vitro and animal studies; the experimental models and level of evidence for each domain are reported in Table 1, and the corresponding clinical evidence is graded in Section 4.4. The bottom box indicates hypothesized, not established, clinical effects. SPM = specialized pro-resolving mediator; TME = tumor microenvironment; PGE2 = prostaglandin E2; PPAR-γ = peroxisome proliferator-activated receptor gamma; MuRF1 = muscle RING finger 1; Atrogin-1 = muscle atrophy F-box protein; PGC-1α = peroxisome proliferator-activated receptor gamma coactivator 1-alpha.

**Figure 2:**
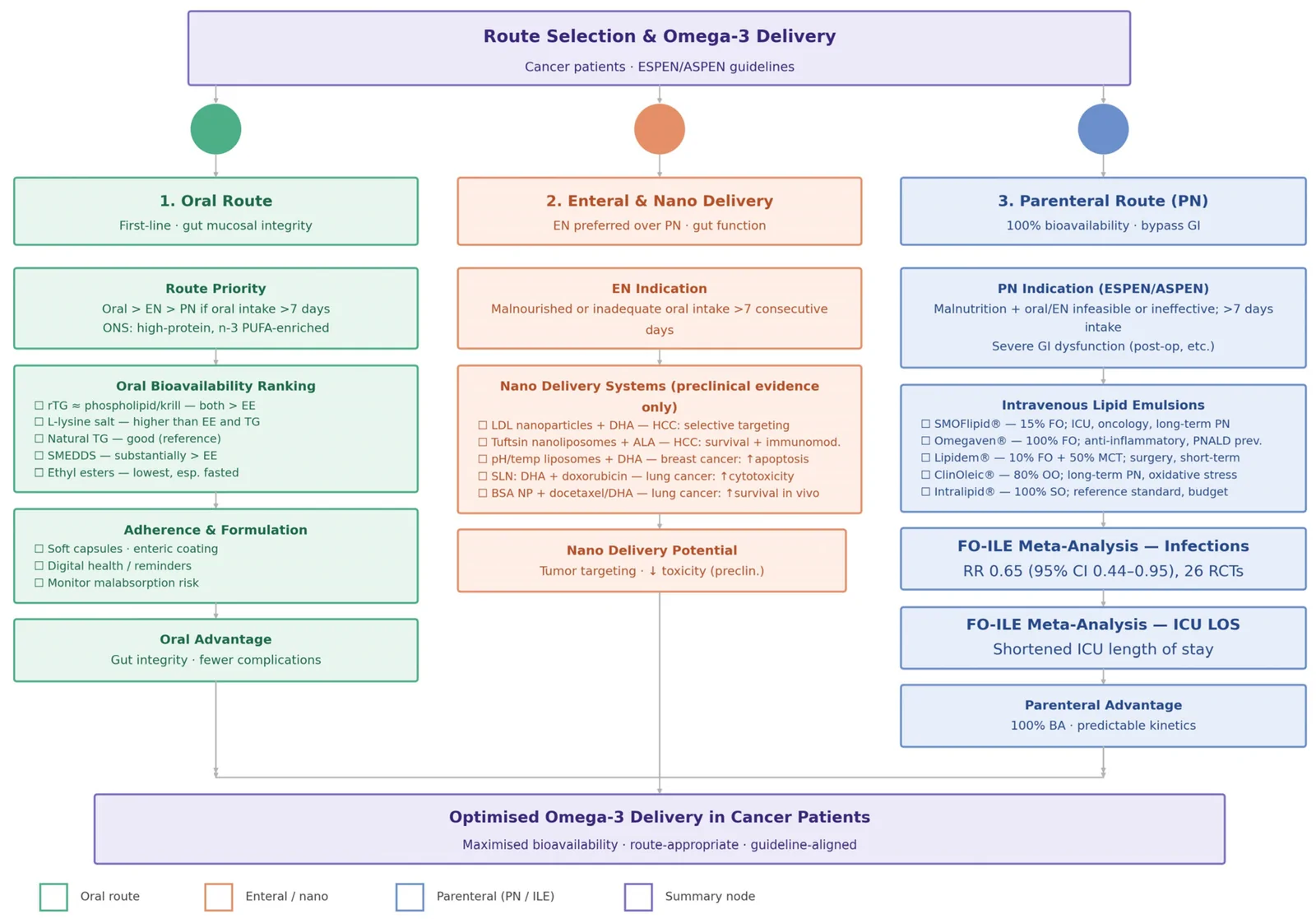
Routes of administration and bioavailability of omega-3 fatty acids in cancer patients. Oral, enteral, and parenteral routes are hierarchically prioritized per ESPEN/ASPEN guidelines. Oral formulations are ranked by intestinal bioavailability. Nanotechnology-based delivery systems are preclinical only. ILE formulations ranked by fish oil content. rTG = re-esterified triglyceride; EE = ethyl esters; SMEDDS = self-microemulsifying drug delivery system; ILE = intravenous lipid emulsion; FO = fish oil; BA = bioavailability; ONS = oral nutritional supplement; HCC = hepatocellular carcinoma; SLN = solid lipid nanoparticle; BSA NP = bovine serum albumin nanoparticle.

**Table 1:** Mechanistic targets of EPA and DHA relevant to cancer cachexia and tumor biology, with the principal experimental models and level of evidence supporting each mechanism.

Domain	Molecular Target	Effect of EPA/DHA	Potential Clinical Relevance	Principal Experimental Models	Level of Evidence
Systemic inflammation	NF-κB, PPAR-γ, COX/LOX	NF-κB inhibition; PPAR-γ activation; eicosanoid shift toward less inflammatory series-3/5	Reduced TNF-α, IL-6, IL-1β; lower catabolic drive	Human endothelial and immune cell lines in vitro [13,17]; RCTs in lung cancer measuring circulating TNF-α, IL-6, CRP [18,19]	Preclinical + human biomarker (surrogate) data
Inflammation resolution	SPMs (resolvins, protectins, maresins)	Active pro-resolution signaling without immunosuppression	TME remodeling; improved immunosurveillance (hypothesized)	Murine models of acute, non-oncological inflammation [20]; tumor models [21,22]	Preclinical only
Tumor proliferation	PI3K/AKT, cyclin D1	Suppression of PI3K/AKT; reduced cyclin D1; cell cycle arrest	Anti-proliferative effect in experimental tumor models	Human breast cancer cell lines (e.g., MCF-7) [23,24,25]; colorectal cancer models [26]	Preclinical (in vitro, xenograft)
Angiogenesis	VEGF, endothelial signaling	VEGF inhibition; reduced endothelial cell proliferation	Impaired tumor vascularization; metabolic deprivation of tumor	Endothelial cell cultures and rodent tumor models [25,27]; circulating VEGF in one single-arm phase II study [28]	Preclinical; limited human biomarker data
Apoptosis	Bcl-2/Bax, caspases, ROS	Bax upregulation; Bcl-2 downregulation; ROS generation; caspase activation	Tumor cell apoptosis (selectivity shown in vitro only)	Glioblastoma [29], breast [30], and prostate [31] cancer cell lines	Preclinical (in vitro)
Autophagy	mTOR, PI3K/Akt/mTOR axis	mTOR suppression; autophagic flux induction	Synergistic with apoptosis; context-dependent tumor cell death	MCF-7 breast [32] and prostate [31] cancer cell lines	Preclinical (in vitro)
Muscle catabolism	Ubiquitin-proteasome (MuRF1, Atrogin-1), NF-κB, myostatin	Downregulation of MuRF1/Atrogin-1; myostatin suppression; NF-κB inhibition	Reduced skeletal muscle proteolysis; lean mass preservation (not consistently shown in patients)	MAC16 adenocarcinoma-bearing mice [33]; orthotopic NSCLC murine model [34]	Preclinical; scarce human muscle data
Muscle anabolism	IGF-1/PI3K/Akt/mTOR, PGC-1α	mTOR anabolic signaling augmentation; PGC-1α upregulation; mitochondrial biogenesis	Enhanced protein synthesis; improved mitochondrial function	Rodent models [35,36]; non-oncological human studies (exercise physiology) [37]	Preclinical + indirect (non-cancer) human data
Chemo/radio sensitization	PGE2, inflammatory microenvironment	PGE2 reduction; anti-inflammatory TME; ROS modulation in tumor	Improved chemotherapy response; reduced mucositis; lower treatment toxicity (small, non-confirmatory studies)	Cancer cell lines and xenografts [38]; small open-label or single-arm clinical studies [39,40,41,28]	Preclinical + low-certainty clinical data

EPA = eicosapentaenoic acid; DHA = docosahexaenoic acid; NF-κB = nuclear factor kappa-light-chain-enhancer of activated B cells; PPAR-γ = peroxisome proliferator-activated receptor gamma; COX = cyclooxygenase; LOX = lipoxygenase; SPM = specialized pro-resolving mediator; TME = tumor microenvironment; TNF-α = tumor necrosis factor alpha; IL = interleukin; PI3K = phosphoinositide 3-kinase; AKT = protein kinase B; VEGF = vascular endothelial growth factor; ROS = reactive oxygen species; mTOR = mechanistic target of rapamycin; MuRF1 = muscle RING finger 1; Atrogin-1 = muscle atrophy F-box protein; IGF-1 = insulin-like growth factor 1; PGC-1α = peroxisome proliferator-activated receptor gamma coactivator 1-alpha; PGE2 = prostaglandin E2; CRP = C-reactive protein; NSCLC = non-small cell lung cancer; RCT = randomized controlled trial. Level of evidence: “Preclinical” = in vitro and/or animal data only; “human biomarker (surrogate) data” = changes in circulating mediators measured in patients, without direct demonstration of a clinical benefit; “low-certainty clinical data” = small, open-label, single-arm, or non-confirmatory clinical studies.

**Table 4:** Principal intravenous lipid emulsion formulations: composition, pharmacological properties, and clinical indications.

Product	Composition	Energy (kcal/L)	Omega-6: Omega-3	Adult Dose	Key Property	Principal Indication
Intralipid^®^ 20%	100% SO	2000	7:1	1.0–2.0 g/kg/day	Reference standard; widely available	General PN; budget-restricted settings
ClinOleic^®^ 20%	80% OO + 20% SO	2000	9:1	1.0–2.0 g/kg/day	Reduced PUFA oxidation; OO-rich	Oxidative stress; long-term PN
Lipidem^®^ 20%	50% MCT + 40% SO + 10% FO	1908	2.7:1	1.0–2.0 g/kg/day	High MCT; moderate FO content	Surgery; short-term anti-inflammatory
SMOFlipid^®^ 20%	30% SO + 30% MCT + 25% OO + 15% FO	2000	2.5:1	1.0–2.0 g/kg/day	Balanced multi-lipid; optimal omega-3 content	ICU; oncology; long-term PN
Omegaven^®^ 10%	100% FO	1120	1:8 (high *n*-3)	0.1–0.2 g/kg/day	Pure EPA+DHA source; hepatoprotective; antioxidant (α-tocopherol)	Add-on anti-inflammatory therapy; parenteral oncology nutrition; PNALD prevention

SO = soybean oil; OO = olive oil; MCT = medium-chain triglycerides; FO = fish oil; PN = parenteral nutrition; PUFA = polyunsaturated fatty acid; EPA = eicosapentaenoic acid; DHA = docosahexaenoic acid; ICU = intensive care unit; PNALD = parenteral nutrition-associated liver disease.

**Table 5:** Selected clinical studies and meta-analyses of omega-3 supplementation in oncological patients, grouped by tumor type and reporting design, cachexia definition, fatty acid composition, dose, formulation, and route.

Study (Year)	Design; Population/*n*	Cachexia Definition/ Nutritional Status	Intervention (Fatty Acid; Dose; Formulation; Route)	Duration	Primary Endpoint	Key Findings
Lung cancer (NSCLC)						
Murphy et al. (2011) [41]	Open-label, SOC-controlled; NSCLC; *n* = 46 vs. standard care	Not an inclusion criterion	EPA+DHA; Fish oil 2.5 g EPA+DHA/day; oral + chemotherapy	Chemo duration	Chemotherapy response rate and clinical benefit	Response rate 60.0% vs. 25.8% in controls (*p* = 0.008); clinical benefit 80.0% vs. 41.9% (non-randomized comparison)
Finocchiaro et al. (2012) [19]	Double-blind RCT; Advanced NSCLC; *n* = 33	Not an inclusion criterion	EPA+DHA capsules; oral; EPA+DHA vs. placebo	Chemo cycle	Inflammatory and oxidative markers	Reduced TNF-α, IL-6, and oxidative stress markers; LBM not assessed
Cheng et al. (2021) [18]	Double-blind RCT; Lung cancer at nutritional risk; *n* = 60	Nutritional risk (screening-based); cachexia not required	EPA+DHA; EPA 1.6 g + DHA 0.8 g/day vs. placebo; oral	12 weeks	Body weight, albumin, CRP, TNF-α	Significant improvements in weight, albumin, CRP, TNF-α vs. placebo
van der Meij et al. (2010) [94]	Double-blind RCT; stage III NSCLC during chemoradiotherapy; *n* = 40	Not an inclusion criterion	EPA+DHA; EPA 2.0 g + DHA 0.9 g/day in protein-energy ONS vs. isocaloric ONS; oral	5 weeks	Body weight; fat-free mass	Better preservation of body weight and fat-free mass vs. control
Gastrointestinal and pancreatic cancer						
Fearon et al. (2003) [95]	Double-blind RCT; advanced pancreatic cancer; *n* = 200	Unintentional weight loss (cachexia)	EPA-predominant; ~2.2 g EPA/day in protein-energy ONS vs. isocaloric ONS; oral	8 weeks	Body weight; LBM	No significant difference in the intention-to-treat analysis; weight and LBM gain correlated with plasma EPA incorporation in compliant patients
Ryan et al. (2009) [96]	Double-blind RCT; esophagectomy for esophageal cancer; *n* = 53	Not required (perioperative setting)	EPA-predominant; 2.2 g EPA/day in EN vs. standard EN; oral pre-operatively, then enteral	5 days pre- to 21 days post-operatively	LBM	LBM preserved with EPA-enriched EN vs. loss in controls
Aoyama et al. (2019) [97]	Multicenter phase III RCT; Gastric cancer post-gastrectomy; *n* = 123	Not required (surgical setting)	EPA-predominant; EPA-enriched ONS (ProSure^®^ 2.2 g EPA/day) vs. standard diet; oral	3 months post-surgery	LBM by BIA at 1 and 3 months	No significant LBM difference at either timepoint (*p* = 0.794; *p* = 0.393)
Eltweri et al. (2019) [28]	Single-arm phase II with historical controls; Advanced esophago-gastric adenocarcinoma; *n* = 20	Not required	EPA+DHA; fish oil ILE; IV Omegaven^®^ weekly + EOX chemo	Chemo duration	Radiological response; inflammatory biomarkers	Partial response 73% vs. 39% in historical controls (*p* = 0.03); reduced IL-2, TNF-α, VEGF; lower grade 3–4 toxicity
Breast cancer						
Douglas et al. (2025)—DHA WIN trial [98]	Placebo-controlled RCT; Non-metastatic breast cancer; *n* = 49	Not required (non-cachectic)	DHA alone; DHA 4.4 g/day during neoadjuvant chemo; oral	Chemo duration	Tolerability and QoL (EORTC QLQ-C30)	DHA supplementation did not significantly affect QoL or exercise behavior versus placebo during chemotherapy
Mixed cancer populations						
Jatoi et al. (2004) [99]	Three-arm RCT; advanced incurable cancer (mainly lung and GI); *n* = 421	Cancer-associated weight loss and/or reduced intake	EPA-predominant; ~2.2 g EPA/day in ONS vs. megestrol acetate vs. both; oral	—	≥10% weight gain	EPA supplement not superior to megestrol acetate; no additional benefit in combination
Fearon et al. (2006) [100]	Double-blind RCT; advanced GI or lung cancer; *n* = 518	Weight loss ≥5%	EPA alone; EPA diester 2 g or 4 g/day vs. placebo; oral	8 weeks	Body weight; LBM	No significant benefit of either dose on weight or LBM vs. placebo
Meta-analyses						
Chua et al. (2024)—Meta-analysis [40]	Meta-analysis of RCTs; Advanced NSCLC with cachexia; pooled RCTs	Cachexia (definitions varied across trials)	EPA+DHA; mixed formulations; oral; ≥2 g EPA+DHA/day, ≥6 weeks (subgroup analyses)	≥6 weeks	Body weight; QoL (EORTC QLQ-C30)	Significant weight gain; improved Physical Functioning and Global Health; LBM change not significant; few, small, heterogeneous trials; dose/duration threshold derived from subgroup analyses and not prospectively validated
Ghoreishy et al. (2024)—Meta-analysis [101]	Dose–response meta-analysis of RCTs; Mixed oncological; 33 RCTs, *n* = 2047	Mixed (cachexia not required in all trials)	EPA, DHA, or both; Variable omega-3 dose vs. placebo; mixed formulations and routes	Variable	Body weight and composition	Dose-dependent weight gain per +1 g/day up to 4 g/day (SMD 0.52 kg); LBM, fat mass, BMI not significant; *I*^2^ = 79–97%

NSCLC = non-small cell lung cancer; LBM = lean body mass; BIA = bioelectrical impedance analysis; RCT = randomized controlled trial; EPA = eicosapentaenoic acid; DHA = docosahexaenoic acid; ONS = oral nutritional supplement; CRP = C-reactive protein; TNF-α = tumor necrosis factor alpha; IL-6 = interleukin-6; VEGF = vascular endothelial growth factor; IV = intravenous; QoL = quality of life; EORTC QLQ-C30 = European Organisation for Research and Treatment of Cancer Quality of Life Questionnaire Core 30; SMD = standardized mean difference; SOC = standard of care; EN = enteral nutrition; ILE = intravenous lipid emulsion; GI = gastrointestinal; EOX = epirubicin, oxaliplatin, capecitabine. Studies are grouped by tumor type to allow a stratified reading (see Section 4.5).

**Table 6:** Consistency of the clinical evidence on omega-3 PUFA supplementation in patients with cancer, by outcome.

Outcome	Direction and Consistency of Effect	Main Supporting Evidence [Ref.]	Main Null or Conflicting Evidence [Ref.]	Overall Appraisal
Circulating inflammatory biomarkers (CRP, IL-6, TNF-α)	Reduction reported in most trials that measured them	Small RCTs in lung cancer [18,19]; phase II study in esophago-gastric cancer [28]; meta-analysis [42]	Heterogeneous assays, sampling times, and populations	Consistent (surrogate endpoint)
Body weight	Small gains or stabilization in pooled analyses; inconsistent across individual RCTs	Meta-analyses [40,101,111]; RCTs [18,94]	Large double-blind RCTs [95,99,100]	Suggestive (driven by meta-analyses)
Lean body mass	Preservation in some small trials; no significant pooled effect	Small RCTs [94,96]	Meta-analyses [40,101]; RCTs [95,97,100]	Inconclusive
Quality of life (EORTC QLQ-C30)	Improvement in Physical Functioning and Global Health, mainly in advanced NSCLC	Meta-analysis [40]	DHA WIN trial in breast cancer [98]; fatigue and appetite domains inconsistently reported	Suggestive (NSCLC; selected domains)
Chemotherapy tolerability and toxicity	Fewer adverse events reported in small studies	Open-label or single-arm studies [28,41]	No adequately powered RCT; heterogeneous toxicity reporting	Suggestive, low certainty
Tumor response	Higher response rates in small non-randomized studies	[28,41]	No confirmatory RCT	Inconclusive (hypothesis-generating)
Overall survival	Associations reported mainly in secondary or exploratory analyses	[43]	Trials not powered for survival; short follow-up	Inconclusive
Appetite and food intake	Weak guideline recommendation based on low-level evidence	ESPEN guideline [92]	ASCO guideline [93]; Cochrane review [107]	Inconclusive (low certainty)

Consistent = concordant direction of effect across individual trials and pooled analyses; suggestive = favorable pooled estimates not consistently reproduced in individual trials, or data restricted to specific populations or domains; inconclusive = conflicting, null, or insufficient data. CRP = C-reactive protein; IL-6 = interleukin-6; TNF-α = tumor necrosis factor alpha; RCT = randomized controlled trial; NSCLC = non-small cell lung cancer; EORTC QLQ-C30 = European Organisation for Research and Treatment of Cancer Quality of Life Questionnaire Core 30; ESPEN = European Society for Clinical Nutrition and Metabolism; ASCO = American Society of Clinical Oncology.

**Table 7:** Stratified interpretation of the clinical evidence according to the main sources of heterogeneity.

Stratification Factor	Categories Studied	Stratified Findings	Implications for Interpretation and Future Trials
Fatty acid composition	EPA alone or EPA-predominant [95,96,99,100]; DHA alone [98]; EPA+DHA [18,41,19,94]	Large EPA/EPA-predominant trials in mixed or pancreatic cancer negative [95,99,100], except LBM preservation after esophagectomy [96]; DHA alone: no QoL benefit in breast cancer [98]; EPA+DHA: most favorable NSCLC data [18,40,94]	Do not extrapolate across preparations; report the EPA:DHA ratio [112]
Dose	<2 g/day; 2–4 g/day; >4 g/day	Dose–response for body weight up to 4 g/day [101]; no benefit of either 2 or 4 g/day EPA vs. placebo [100]	Dose-finding trials needed; ≥2 g/day is a pragmatic, not validated, reference
Formulation and route	Capsules (EE/TG); EPA-enriched protein-energy ONS; EPA diester; EN; IV fish oil ILE	ONS effects confounded by energy and protein; IV data small and uncontrolled [28]; micellar and nanoformulations not yet tested clinically	Analyze by vehicle; use isocaloric controls; verify EPA/DHA incorporation
Duration	<6 weeks (perioperative); 6–12 weeks; >12 weeks	Short perioperative courses mostly null [97] (exception: [96]); NSCLC signal at ≥6 weeks [40]; few trials >12 weeks	Minimum 12-week intervention and follow-up
Cancer type and setting	NSCLC (chemotherapy); GI/pancreatic (surgery, palliative care); breast (neoadjuvant); mixed	NSCLC: most favorable; GI: highest nutritional need, mixed results [97,28,95,96]; breast: null [98]; mixed: negative [100,99]	Tumor-specific interpretation; stratify or restrict by tumor type
Cachexia definition and stage	Pre-2011 weight-loss thresholds; 2011 consensus [3]; nutritional risk screening; unselected	Stage rarely pre-specified; benefit in early vs. refractory cachexia untested	Adopt the consensus definition; pre-specify stage as a stratification variable

EPA = eicosapentaenoic acid; DHA = docosahexaenoic acid; LBM = lean body mass; QoL = quality of life; NSCLC = non-small cell lung cancer; EE = ethyl ester; TG = triglyceride; ONS = oral nutritional supplement; EN = enteral nutrition; IV = intravenous; ILE = intravenous lipid emulsion; GI = gastrointestinal.

## Data Availability

No new data were created or analyzed in this study. Data sharing is not applicable to this article.

## Abbreviations

The following abbreviations are used in this manuscript:

Abbreviation	Full Term
ALA	alpha-linolenic acid
ASCO	American Society of Clinical Oncology
ASPEN	American Society for Parenteral and Enteral Nutrition
BSA	bovine serum albumin
DHA	docosahexaenoic acid
EE	ethyl ester
EN	enteral nutrition
EORTC QLQ-C30	European Organisation for Research and Treatment of Cancer Quality of Life Questionnaire Core 30
EPA	eicosapentaenoic acid
ESMO	European Society for Medical Oncology
ESPEN	European Society for Clinical Nutrition and Metabolism
FO	fish oil
HPN	home parenteral nutrition
IGF-1	insulin-like growth factor 1
IL-6	interleukin-6
ILE	intravenous lipid emulsion
LBM	lean body mass
MCT	medium-chain triglycerides
mTOR	mechanistic target of rapamycin
MuRF1	muscle RING finger 1
NF-κB	nuclear factor kappa-light-chain-enhancer of activated B cells
NLC	nanostructured lipid carrier
NSCLC	non-small cell lung cancer
ONS	oral nutritional supplement
OO	olive oil
PGC-1α	peroxisome proliferator-activated receptor gamma coactivator 1-alpha
PGE2	prostaglandin E2
PIF	proteolysis-inducing factor
PN	parenteral nutrition
PPAR-γ	peroxisome proliferator-activated receptor gamma
PUFA	polyunsaturated fatty acid
RCT	randomized controlled trial
rTG	re-esterified triglyceride
SLN	solid lipid nanoparticle
SMEDDS	self-microemulsifying drug delivery system
SO	soybean oil
SPM	specialized pro-resolving mediator
TG	triglyceride
TME	tumor microenvironment
TNF-α	tumor necrosis factor alpha
UPS	ubiquitin-proteasome system
VEGF	vascular endothelial growth factor
